# A Machine Learning Model for Diagnosing Opportunistic Infections in HIV Patients: Broad Applicability Across Infection Types

**DOI:** 10.1111/jcmm.70497

**Published:** 2025-03-23

**Authors:** Hao Chen, Fanxuan Chen, Yijun Wang, Enna Cai, Wangzheng Pan, Yichen Li, Zefei Mo, Hao Lou, Chufan Ren, Chenyue Dai, Xingbo Shan, Hui Ye, Zhenwei Xu, Pu Dong, Han Zhou, Shuya Xu, Tianye Zhu, Mingzhi Su, Xingguo Miao, Xiaoqu Hu, Liang Hong, Yi Wang, Feifei Su

**Affiliations:** ^1^ Department of Infectious Diseases Wenzhou Central Hospital Wenzhou China; ^2^ The First School of Medicine, School of Information and Engineering Wenzhou Medical University Wenzhou China; ^3^ School of Biomedical Engineering, School of Ophthalmology and Optometry, Eye Hospital Wenzhou Medical University Wenzhou China; ^4^ The Second Clinical Medical College of Wenzhou Medical University Wenzhou China; ^5^ The School of Nursing, Wenzhou Medical University Wenzhou China; ^6^ Wenzhou Medical University Renji College Wenzhou China; ^7^ School of Computer Science and Software Engineering University of Science and Technology Liaoning Anshan China; ^8^ Department of Infectious Diseases Wenzhou Sixth People's Hospital Wenzhou China; ^9^ Department of Infectious Diseases Taishun County People's Hospital Wenzhou China; ^10^ Department of Infectious Diseases The Third Affiliated Hospital of Wenzhou Medical University Wenzhou China; ^11^ Department of Infectious Diseases Wencheng People's Hospital Wenzhou China; ^12^ Zhejiang Industry Polytechnic College Shaoxing China; ^13^ Department of Surgical Oncology The First Affiliated Hospital of Wenzhou Medical University Wenzhou China; ^14^ Wenzhou Key Laboratory of Diagnosis and Treatment of Emerging and Recurrent Infectious Diseases Wenzhou China

**Keywords:** AIDS, diagnostic model, HIV, machine learning, opportunistic infections

## Abstract

Opportunistic infections (OIs) are the leading cause of hospitalisation and mortality among Human Immunodeficiency Virus‐infected (HIV‐infected) patients. The diverse pathogen types and intricate clinical manifestations associated present a formidable challenge to the timely diagnosis of these infections. This study aims to use machine learning techniques to develop a diagnostic model that quickly identifies whether HIV‐infected patients have any type of OIs, without being limited to specific infections, thus adapting to various clinical scenarios. This study is a retrospective cohort study that collected clinical data from HIV‐infected patients at four healthcare organisations in China. A total of twelve machine learning classification algorithms were employed for the purposes of model training and evaluation. Additionally, feature reduction was conducted through the implementation of an importance ranking, with the objective of eliminating any redundant features. In conclusion, both the five features based on Shapley additive explanations (procalcitonin, haemoglobin, lymphocyte, creatinine, platelet) and the five features based on Permutation Importance explanations (procalcitonin, lymphocyte, haemoglobin, creatinine, indirect bilirubin) achieved the highest F1 score when evaluated using the adaptive boosting classifier model. The scores on the test set were 0.9016 and 0.9063, respectively, which significantly outperformed the best 32‐feature model, gradient boosting classifier, which had a test set F1 score of 0.8991.

## 1 Introduction

Opportunistic infections (OIs) are serious infections caused by pathogens that normally do not cause disease in healthy individuals but can lead to infections in immunocompromised individuals [1]. Human Immunodeficiency Virus (HIV) primarily targets the human immune system, leading to a significant decline in CD4+ T cells, which profoundly affects immune function, particularly cellular immunity [2]. As the number of CD4 + T cells declines, the risk of OIs increases remarkably. Consequently, the prevalence of OIs among HIV‐infected patients is markedly higher than that of the general population, particularly among those in the advanced stages of Acquired Immune Deficiency Syndrome (AIDS). A multicohort study found a sevenfold higher OIs prevalence in HIV‐infected patients than in the general population with an initial CD4 count below 200 cells/mm^3^ [3]. Prior to the advent of antiretroviral therapy (ART), HIV‐infected patients typically developed HIV‐associated OIs approximately 7–10 years after initial infection, with a median survival time of only 9 months once OIs were diagnosed [4]. ART has significantly improved the prognosis of HIV‐infected patients, reducing the incidence of OIs and extending life expectancy by effectively suppressing viral replication. However, despite these benefits, OIs remain a major cause of hospitalisation and mortality among HIV‐infected individuals, particularly in low‐ and middle‐income countries [5, 6]. An Ethiopian study demonstrated that children with OIs exhibited a 2.5‐fold increased risk of mortality compared to those without OIs [7].

AIDS‐related OIs present with complex and nonspecific clinical manifestations, involve diverse pathogens, and may affect multiple organ systems [8, 9, 10]. Patients often suffer from multiple infections simultaneously, and many are only diagnosed with AIDS after OIs have occurred, delaying early diagnosis and treatment [11]. A spectrum analysis of OIs in hospitalised AIDS patients revealed the wide variety of infections commonly seen in this population, with the most prevalent being Pneumocystis pneumonia, occurring in 42.1% of cases, followed by *Mycobacterium tuberculosis* (31.4%), cytomegalovirus (20.9%), cryptococcosis (9.0%) and *Mycobacterium avium* complex (5.2%) [12]. At present, the diagnosis of OIs is primarily based on the patient's clinical symptoms, laboratory tests (such as blood cultures and serology) and pathogen detection [13]. However, these methods have limitations in terms of sensitivity and specificity. For example, blood cultures, while important for pathogen identification, can take several days and may not detect slow‐growing or fastidious pathogens, and serology may not differentiate between past infections and active disease. As a result, high‐risk patients may fail to receive timely intervention [14]. The emergence of molecular biology techniques (such as PCR testing), which detect the deoxyribonucleic acid (DNA) or ribonucleic acid (RNA) of pathogens, has provided a highly sensitive and rapid diagnostic method [15]. However, the high cost and complexity of these techniques limit their widespread use in resource‐limited areas [16]. Therefore, the development of effective diagnostic tools for HIV co‐morbid OIs is particularly urgent.

The advent of machine learning has ushered in a new era of disease prediction and diagnosis [17, 18, 19]. Machine learning models can integrate complex data, including demographic information, past medical history, laboratory results (e.g., blood cultures, PCR tests and serology) and imaging tests, to generate individualised infection risk assessments and diagnostic recommendations [20, 21, 22, 23, 24]. Furthermore, they can continuously improve diagnostic accuracy (ACC) through a self‐iterative process, which shows great potential in the diagnosis of AIDS‐related OIs. For example, Chagas et al. (2024) developed a predictive model to assist in the diagnosis of AIDS‐associated Pneumocystis jirovecii pneumonia, with ACC, precision, recall and the area under the curve (AUC) scores exceeding 0.8. Nevertheless, the present utilisation of machine learning in OIs remains confined to particular infection types, exhibiting an inadequate capacity for generalisation in addressing complex and diverse infections [25, 26]. Moreover, there has been a paucity of diagnostic studies conducted, with a greater emphasis placed on prognosis prediction and new drug development [27, 28, 29, 30]. This study aims to develop a diagnostic model that can identify whether HIV‐infected patients are co‐infected with various types of OIs by analysing the clinical data of HIV patients using machine learning algorithms to adapt to the specific needs of different clinical scenarios.

## 2 Method

### 2.1 Data Sources and Study Population

**FIGURE 1:**
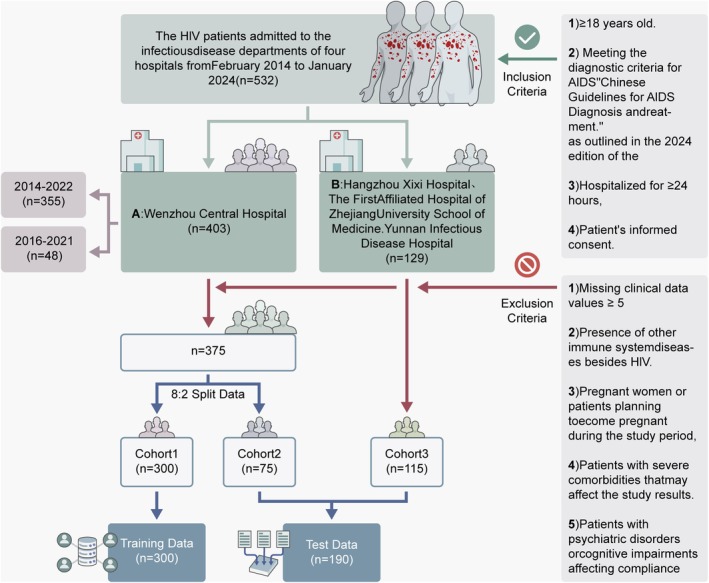
Flowchart of data inclusion and exclusion.

This study was a retrospective cohort study that strictly adhered to the principles set forth in the Declaration of Helsinki. The study protocol was conducted following approval by the Ethics Committee of Wenzhou Central Hospital, the Ethics Committee of Hangzhou Xixi Hospital, the Ethics Committee of the First Hospital Affiliated to the Medical College of Zhejiang University, and the Ethics Committee of Yunnan Provincial Hospital of Infectious Disease. Clinical data on HIV patients from four Chinese healthcare institutions were collected in accordance with the stipulations of the inclusion and exclusion criteria (Figure 1). In accordance with the established inclusion criteria, data from 403 patients were incorporated into the study. These patients were drawn from Wenzhou Central Hospital, comprising 355 patients from 2014 to 2022 and 48 patients from 2016 to 2021. Additionally, 129 patients from 2016 to 2021 were included from Hangzhou Xixi Hospital, the First Affiliated Hospital of Zhejiang University School of Medicine, and Yunnan Provincial Hospital of Infectious Disease. Following the application of exclusion criteria, a total of 42 patients were excluded from the group. Of these, 28 patients were excluded from Wenzhou Central Hospital, while a further 14 patients were excluded from the other three medical centres. Ultimately, 375 patients were included in Wenzhou Central Hospital (Cohort 1 and Cohort 2), and 115 patients were included in the other three medical centres (Cohort 3). A standardised baseline table was designed for the integration of data on patient characteristics, including demographic factors, clinical symptoms, laboratory findings and radiological characteristics (Table S1).

### 2.2 Clinical Data Collection

This study employed a systematic methodology for the collection and analysis of clinical data. First, we searched the PubMed database for literature on HIV‐associated OIs published between 2010 and 2024. The search keywords included ‘HIV’ ‘AIDS’ ‘OIs’ ‘clinical features’ and ‘risk factors’ Through screening, 32 key features commonly used to determine co‐morbid OIs in HIV patients were identified (Table S2). To guarantee the precision and clinical significance of the categorisation, we classified and diagnosed HIV co‐infections in accordance with the most recent recommendations of the Centres for Disease Control and Prevention (CDC). Patients within each cohort were classified as either co‐infected or unco‐infected based on their infection status.

### 2.3 Data Preprocessing and Statistical Analysis

The data preprocessing in this study was conducted using Python 3.10.11, and all data were retained to four decimal places. The ACC of the data was verified and confirmed individually by three senior clinicians (with 24, 11 and 6 years of service, respectively) in the Department of Infection of Wenzhou Central Hospital. In the categorical variables, a value of ‘0’ indicates an asymptomatic state, while a value of ‘1’ indicates a symptomatic state. In the gender variables, a value of ‘0’ indicates a female subject, while a value of ‘1’ indicates a male subject.

In the event of missing data, the column means of the same group of data were used to interpolate missing values of continuous variables. Categorical variables were uniformly filled in as ‘0’ with no missing values for gender. It should be noted that HIV patients may exhibit extreme values in serological indicators due to immunodeficiency. To mitigate the influence of extreme values on the analysis, the continuous variables were transformed using the natural logarithm function (i.e., ln(x + 1)). Subsequently, Z‐standardisation was implemented to unify the dimensions, enabling the model to address disparate characteristics in a more balanced manner.

Ultimately, the data from Wenzhou Central Hospital were randomly divided into 8:2 ratios, of which 80% (*n* = 300) were used as the training set for model building and parameter tuning, and the remaining 20% (*n* = 75) were merged with the data from the other three hospitals to form the test set (*n* = 190). The test set derived from multicentre data was conducive to a realistic evaluation of the model's predictive performance and generalisation ability.

To ascertain the discrepancies in the distributions of variables within and between the train and test sets, we employed the Mann–Whitney U test for continuous variables and the Pearson chi‐square test for categorical variables. The significance level for all statistical tests was set at *p* < 0.05.

Furthermore, we conducted a correlation analysis between the features, measuring the correlation between continuous variables with the Pearson correlation coefficient and assessing the correlation between categorical variables with the Phi coefficient.

### 2.4 Model Selection

In order to address the possible bias in feature selection caused by a single machine learning algorithm, this study selected twelve representative classification algorithms, including adaptive boosting classifier (AdaBoost Classifier), gradient boosting classifier (Gradient Boosting Classifier), decision tree classifier (Decision Tree Classifier), ridge classifier with cross‐validation (Ridge Classifier CV), stochastic gradient descent classifier (SGD Classifier), passive‐aggressive classifier (Passive Aggressive Classifier), Linear support vector classifier (Linear SVC), support vector classifier (SVC), bernoulli naive bayes classifier (BernoulliNB), extremely randomised trees classifier (Extra Tree Classifier), random forest classifier (Random Forest Classifier) and perceptron. These algorithms were implemented using the scikit‐learn library (version 1.5.1) in the Python 3.12.5 environment.

### 2.5 Model Training and Evaluation

In this study, we employed a precise machine learning model optimisation strategy to guarantee that the model demonstrated robust generalisation ability on the test set. An exhaustive hyperparameter grid was initially defined, and a grid search method was subsequently employed to conduct a meticulous evaluation of parameter combinations. To quantify the performance of each parameter configuration, we used a five‐fold cross‐validation method, which ensured comprehensive and fair evaluation by dividing the train set into five parts, using four of them for model training in turn, and the remaining part for internal validation. Given the imbalanced distribution of positive and negative samples in our dataset, we devoted particular attention to the optimisation of the F1 score in each round of cross‐validation.

During the grid search, the mean F1 score in the five‐fold cross‐validation for each parameter combination was recorded. Once a parameter combination was identified as significantly enhancing the average F1 score, it was regarded as the current optimal parameter configuration. The optimal parameter combination was then employed for training the model on the entire train set. To further validate the model's generalisation ability, AUC, ACC and F1 Score on the train set and test set were output for analysis.

### 2.6 Feature Selection and Model Reduction

In this study, we employed a systematic feature selection process with the objective of streamlining the feature set of the machine learning model while maintaining its predictive performance, thereby developing a cost‐effective and more generalisable diagnostic model. To achieve feature simplification, we initially evaluated the importance of 32 features in twelve distinct machine learning models, employing two methodologies: shapley additive explanations (SHAP) and permutation importance (PI). SHAP quantifies the marginal contribution of each feature to model predictions by calculating the Shapley value of each feature [31]. PI confirms the importance of features by randomly shuffling the order of features and monitoring changes in model performance [32].

Considering the aforementioned analyses, we proceeded to generate feature importance rankings for 32 features in each model and selected the top ten ranked features as the initial feature set. Subsequently, the number of features was gradually reduced from ten to five. Following each reduction, hyperparameter optimisation was performed for the new feature set, and the model was retrained and re‐evaluated. This step ensured that the model could rapidly adapt and sustain optimal performance with a reduced number of features.

In this study, we employed the uniform manifold approximation and projection (UMAP) algorithm to perform dimensionality reduction on the training set and test set, respectively, incorporating the original 32 features, 10 features (SHAP), 10 features (PI), 5 features (SHAP) and 5 features (PI), thereby facilitating the visualisation and comparison of sample distributions in low‐dimensional spaces. For each number of features, the model with the best F1 score on the corresponding test set was selected as the optimal model. To further verify the effect of feature selection, we visually displayed the prediction probabilities of the optimal models with 32, 10 and 5 features on the train and test sets, respectively.

## 3 Results

### 3.1 Demographic Results

**FIGURE 2:**
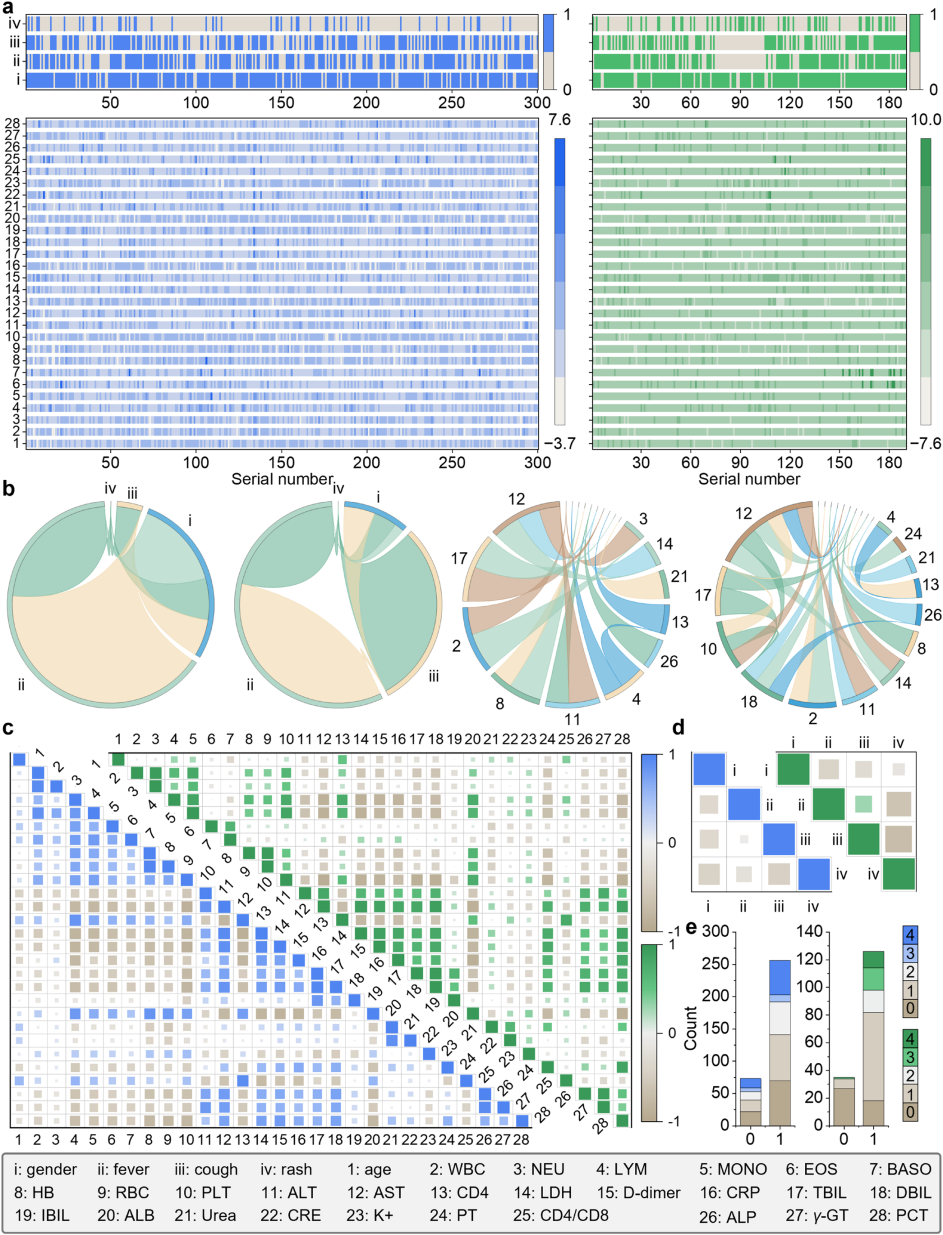
Demographics and feature correlation analysis. (a) Heat map of the distribution of the 4 clinical features belonging to the categorical variables and the 28 clinical features belonging to the continuous variables in the train set (*n* = 300) and in the test set (*n* = 190), respectively. ‘0’ represents asymptomatic and ‘1’ represents symptomatic. The blue colour is the train set and the green colour is the test set, the same below. (b) Correlation chord diagram between categorical and continuous variable features in the training and test sets. Among these 32 features, correlation lines with absolute values of correlation less than 0.5 are not shown. When the absolute value of the correlation exceeds 0.5, the lines connecting the features become thicker as the correlation increases, and the features are arranged clockwise from the 12 o'clock position from smallest to largest percentage. Among them, the part of the features not labelled in the correlation chord diagram of continuous variable features in the train set are 5, 7, 9, 20, 27, 15, 18, 25, 19, 22 in clockwise order, and the part of the features not labelled in the correlation chord diagram of continuous variable features in the test set are 3, 5, 9, 27, 15, 20, 28, 25, 19, 22 in clockwise order. (c) Feature correlation heatmap of continuous variable features in the train and test sets (d) Feature correlation heatmap of categorical variable features in the train and test sets (e) Heatmap of distribution of different types of OIs in patients in the train and test sets.

Figure 2a shows the distribution of 32 clinical characteristics of patients in the train and test sets. The differences in the distribution of variables between and within the train and test sets are shown in Table S3. The results show that infection with pathogens is diverse and complex, with HIV combined with fungal infection being the most common. We further analyse the correlations within different continuous and categorical indicators in the train and test sets (Figure 2b–d). The two most highly correlated features are neutrophils (NEU) and white blood cells (WBC), with Pearson correlation coefficients of 0.9403 and 0.9262 in the train and test sets, respectively. Figure 2e shows the status of HIV patients with combined fungal, bacterial, viral or mixed infections in the train and test sets.

### 3.2 Performance Comparison of Twelve Models With 32 Features

The experiment evaluates the performance of 12 machine learning models on the train and test sets. Using 32 features, ensemble models and Decision Tree models generally perform well on the train set, with Random Forest Classifier, Gradient Boosting Classifier, Extra Trees Classifier and Decision Tree Classifier all having an F1 score of 1, and AdaBoost Classifier having an F1 score of 0.9544. However, the performance of these models on the test set drops significantly, so it can be concluded that these ensemble learning models exhibited overfitting in the case of the 32‐feature dataset. Moreover, the overfitting phenomenon of the nonlinear models is more obvious than that of the linear models. For example, even the Gradient Boosting Classifier model, which performed best on the external test set, had an F1 score of 1 on the train set, but its F1 score on the test set did not exceed 0.90, indicating that its generalisation ability was limited. In contrast, linear models (such as Ridge Classifier CV and SGD Classifier) and support vector machine (SVM) models (SVC and LinearSVC) did not show obvious signs of overfitting, but their F1 scores and other metrics were weaker than those of Ensemble Learning and Decision Tree models.

### 3.3 Result of Feature Selection

**FIGURE 3:**
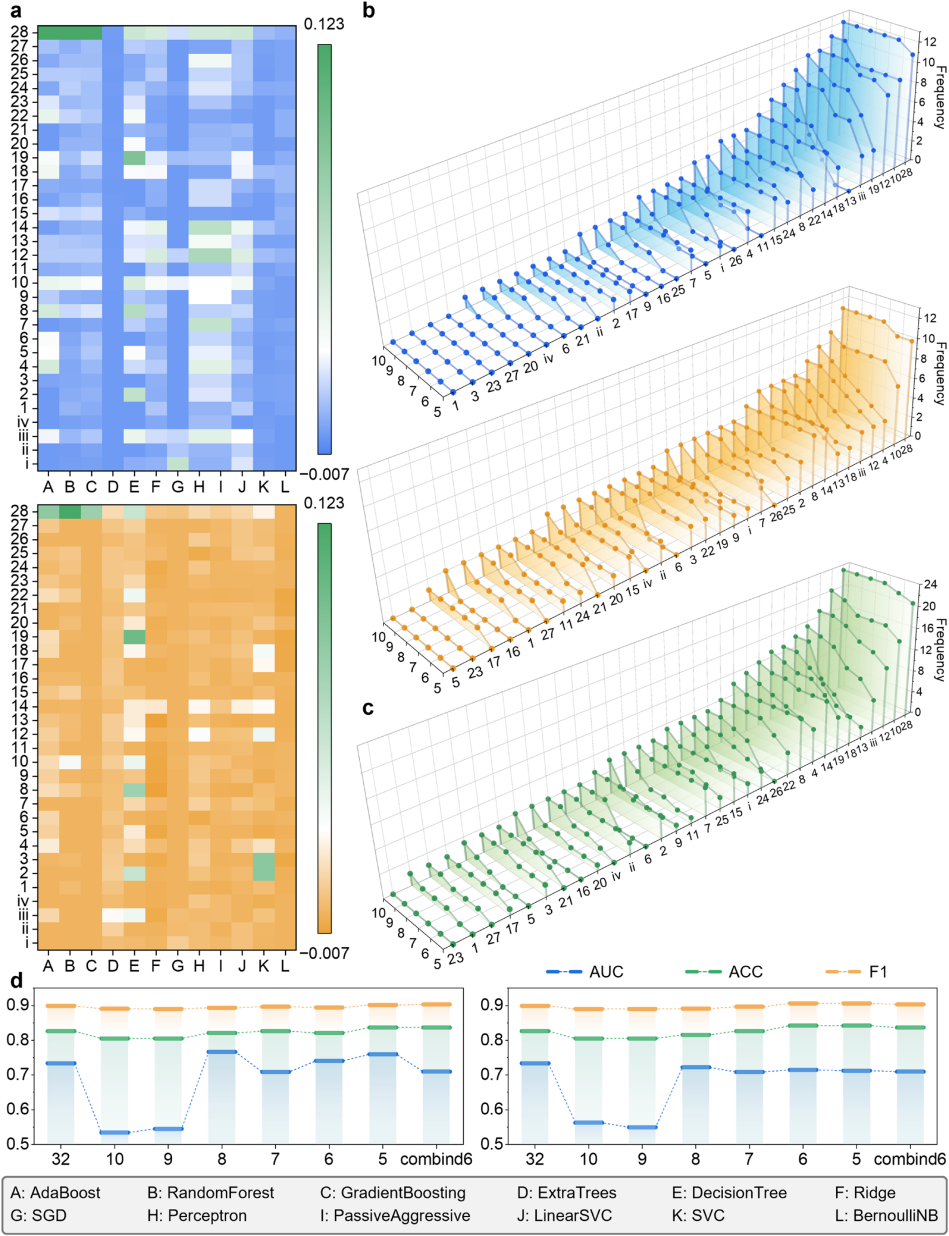
Visualisation of the feature selection results after using the SHAP and PI methods, respectively. (a) Feature importance heatmap of the 32 features in the 12 machine learning models. (b) Frequency of the 32 features ranked in the top 10, 9, 8, 7, 6 and 5 in the 12 machine learning models. (c) The sum of the frequencies of the top 10, 9, 8, 7, 6 and 5 rankings of the 32 features in the 12 machine learning models under the two interpretations of SHAP and PI. (d) The AUC, ACC and F1 changes of the optimal model and the 6 combined features (PCT, LYM, HB, CRE, IBIL and PLT) models during the feature selection process from 10 features to 5 features. The designations of the features are illustrated in Figure 2.

Among the 12 machine learning models, we used both SHAP and PI methods to obtain the contribution scores of 32 features to the models (Figure 3a) and accordingly ranked the features in terms of importance (Figure 3b,c). Although there are some differences in the feature rankings of different models, procalcitonin (PCT) is the feature that appears most frequently in the top 10 most important features, regardless of whether SHAP or PI is used. It appeared in the top 10 most important features of almost all models, except BernoulliNB in the PI framework, and ranks high. Furthermore, platelet (PLT) and aspartate aminotransferase (AST) were also frequently selected in the top 10 most important features in most models. Under the SHAP interpretation, PLT and AST ranked in the top ten in 11 and 10 models, respectively; under the PI interpretation, PLT and AST ranked in the top ten in 9 and 7 models, respectively. In addition, the three characteristics PCT, PLT and AST were still in the top five in terms of frequency of occurrence in the ranking of the importance of characteristics for different models. Under the SHAP interpretation, PCT, PLT and AST ranked in the top five in 11, 9 and 8 models, respectively. Under the PI interpretation, these three ranked in the top five in 10, 6 and 3 models, respectively. These results all indicate that PCT, PLT and AST are significantly associated with the identification of HIV in combination with OIs.

### 3.4 Evaluation of the Best Model

**FIGURE 4:**
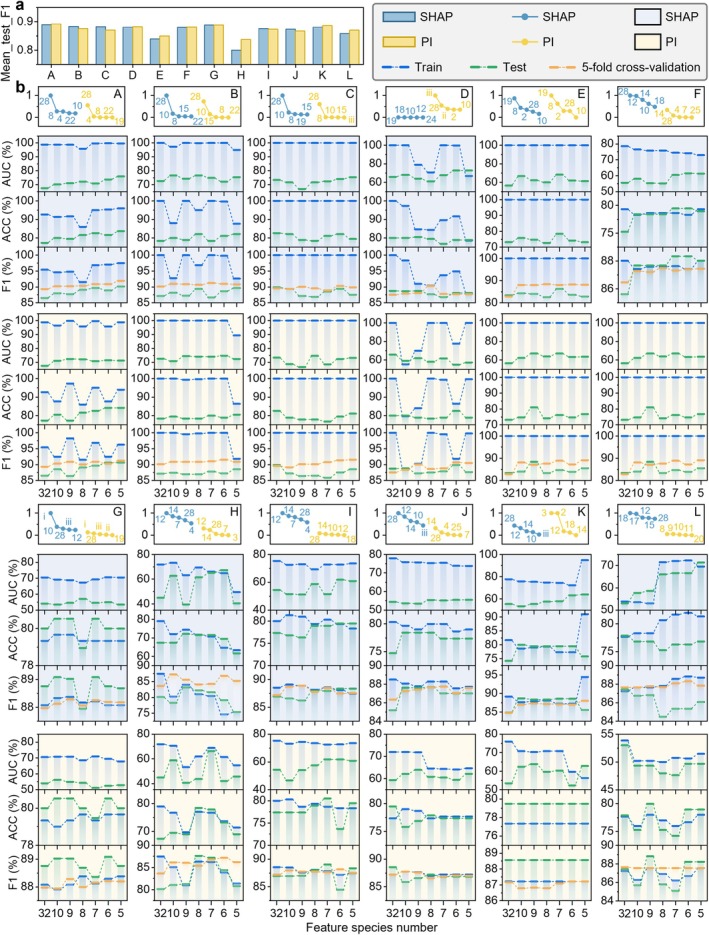
Performance changes of the twelve models during feature selection. (a) Histograms of F1 scores of twelve machine learning models under the two feature importance interpretation methods of SHAP and PI, where F1 Score refers to the combined average of each model in the 10 to 5 feature conditions. (b) Line plots of feature importance and performance variation for the 12 models. The top five feature importance rankings of each model under SHAP and PI interpretations, respectively, are shown in detail, as well as the AUC and ACC values of the models in the train set and test set, and the AUC and ACC values of the models in the five‐fold cross‐validation, the train set and the test set, as the number of features decreases from 32 to 5. Additionally, the trend of F1 score in the train set and test set is illustrated. See Figures 2 and 3 for characterisation and model designation.

Using the top 10, 9, 8, 7, 6 and 5 features for each model, we retrained and tested the model to obtain the AUC, ACC and F1 of the training and test sets, as shown in Figure 4.

In the feature reduction process based on SHAP, the highest F1 score on the test set is 0.9015, which corresponds to the AdaBoost Classifier model and the features PCT, HB, LYM, PLT and CRE. Based on PI, the AdaBoost Classifier model also obtained the highest F1 score of 0.90625 on the test set, and this F1 value was achieved when using six features (PCT, LYM, HB, CRE, IBIL, PLT) and reducing to five features (PCT, LYM, HB, CRE, IBIL). We can see that the optimal model performance under the two feature selection methods is very similar, and both are better than the performance of all models in the 32‐feature test set. Further analysis revealed that the top five feature subset collections of the AdaBoost Classifier model under the SHAP and PI interpretations were PCT, LYM, HB, CRE, IBIL and PLT, and these six features were also the top six features selected by PI.

### 3.5 Visualisation of Results

**FIGURE 5:**
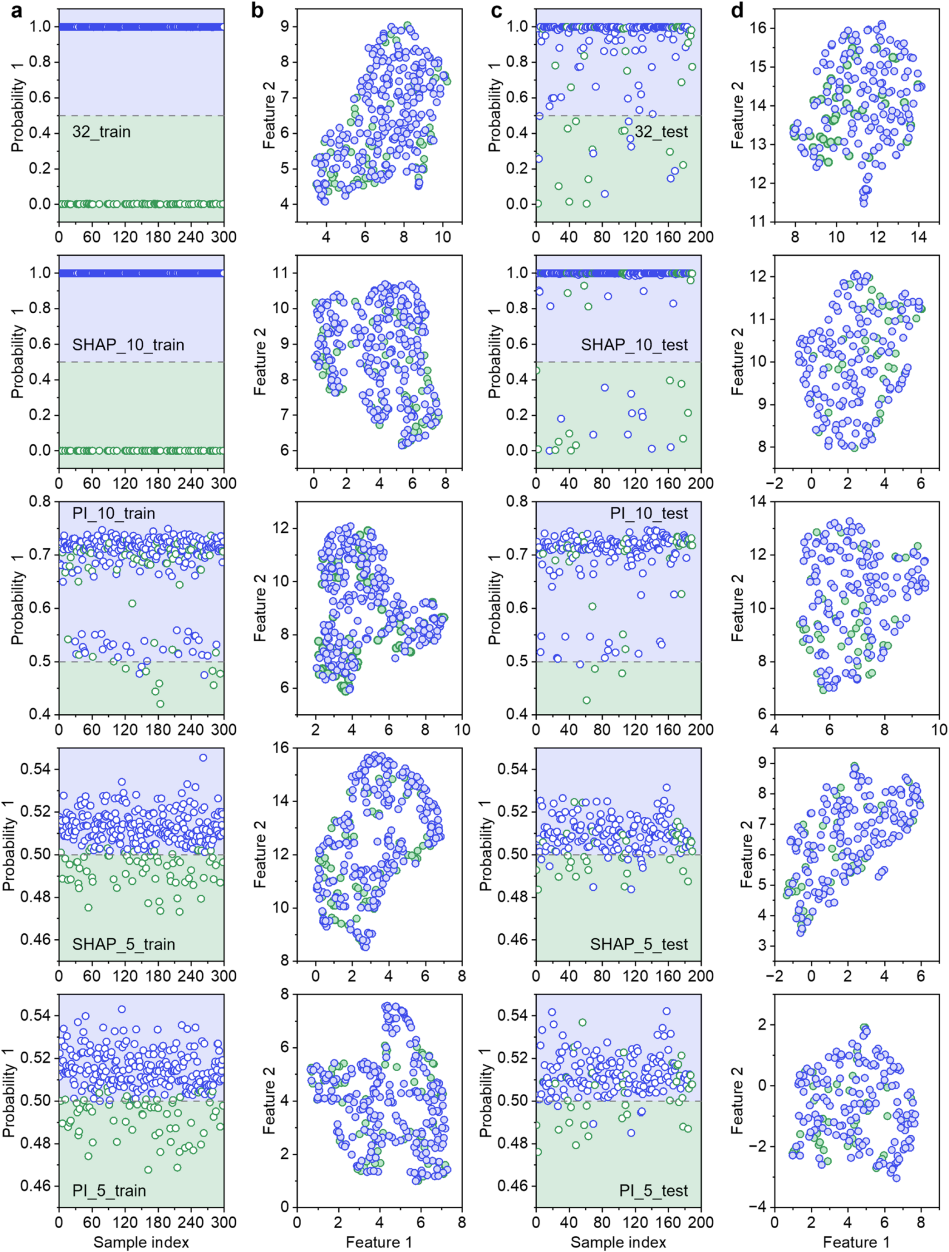
Visualisation of UMAP dimensionality reduction and machine learning fitting. (a) Scatter plot of the prediction probability of the optimal model on the test set for the samples in the train set with different numbers of features. The abscissa is the sample index, and the ordinate prob1 is the probability that the model predicts the sample to be group = 1. The blue dots indicate Group 1, while the green dots indicate Group 0. (b) UMAP dimensionality reduction results of the train set with different number of features (group 0 is HIV patients only, group 1 is HIV patients with OIs). (c) Scatter plot of the prediction probability of the best model in the test set with different numbers of features. (d) UMAP dimensionality reduction results for the test set with different numbers of features.

We use the UMAP algorithm to perform dimensionality reduction on the train set and test set. By finding the neighbourhood relationships of similar data points in high‐dimensional space and projecting them into low‐dimensional space, a two‐dimensional representation of each sample is generated. We visualise the results of the train and test sets after UMAP dimensionality reduction, intuitively displaying and comparing the distribution of the train and test set samples in the low‐dimensional space under different numbers of features (Figure 5). It can be seen that the reduction in the number of features does affect the distribution pattern of the samples. Although the separation between the Group 0 and Group 1 classes is still not obvious, the clustering of similar samples has been enhanced. This result shows that some of the noisy features have been effectively removed during the feature reduction process.

We use a probability scatter plot to further demonstrate the impact of the number of features on the model's prediction performance, where prob1 indicates the probability that the model predicts that a sample belongs to Group 1 (Figure 5 and Figure S1). On the train set, the optimal model (Gradient Boosting Classifier) using all 32 features demonstrates extremely high prediction ACC, with all Group 1 sample points clustered in the prob1 = 1.0 area and all Group 0 sample points clustered in the prob1 = 0 area. However, this performance did not hold true for the test set, where the sample points were more scattered, with no significant classification boundary between Group 0 and Group 1 points. When the number of features is reduced to 10, the predicted probability distributions of the sample points differ between the two explanation methods SHAP and PI (Figure S2). The best model for the test set based on SHAP is Gradient Boosting Classifier, which shows very similar overfitting with 10 features as with 32 features. The optimal model for the test set based on PI is SGD Classifier. Although in the train set, the sample points of Group 0 and Group 1 are not distributed in the two extreme probability regions as in the 32‐feature model, they are mixed in the prob1 probability interval of 0.70 to 0.75; in the test set, the distribution of sample points is also mixed, with no obvious classification boundary. After further reducing the features to 5, the sample points in the train set are no longer overly concentrated in the high probability region but instead show a very clear classification boundary around prob1 = 0.5. Although there are still some mixed sample points in the test set, compared with 32 features and 10 features, the separation effect of Group 0 and Group 1 sample points at the prob1 = 0.5 boundary is significantly enhanced. This series of comparative results shows that after feature selection, the points in Group = 0 and Group = 1 can be better separated, and the separability of the spatial distribution of sample points is enhanced.

## 4 Discussion

AIDS patients often have multiple OIs, including bacterial, fungal and viral infections. The clinical manifestations of these infections are often atypical, and microbiological identification can be time‐consuming, posing a significant challenge to timely diagnosis [33]. The advent of machine learning technology has brought new opportunities for disease prediction and diagnosis [34, 35, 36]. Currently, there are some machine learning models for diagnosing HIV in combination with OIs, but these studies are usually limited to a single type of infection [37]. For example, the model developed by Hu et al. performed well in diagnosing AIDS‐related Talaromyces marneffei, with an AUC of 0.877 [38]. However, in diverse clinical settings, models targeting single infection types struggle to provide comprehensive diagnostic guidance, fail to effectively reduce healthcare costs and time, and may delay optimal treatment. In contrast, this study retrospectively developed an innovative machine learning model with enhanced generalisation ability to rapidly identify the presence of any OI in HIV patients. Its key advantage is broad‐spectrum diagnostic capability, serving as a screening tool to assist in the comprehensive diagnosis of OIs without targeting specific pathogens. This approach significantly reduces the risk of missed diagnoses, improves diagnostic ACC, and provides strong support for early clinical diagnosis and intervention.

In studies involving HIV and OIs, there is a lack of clear diagnostic indicators for multiple infections. As a result, the inclusion criteria and modelling methods for each indicator vary across studies. This study's dataset of 32 correlated diagnostic features complicated machine learning, particularly ensemble models, increasing overfitting risk and limiting generalisation. To address this, we implemented a feature reduction strategy by ranking features based on their importance and removing redundant ones, creating a simplified model with fewer yet more valuable features. This approach is significant not only for the model—reducing the risk of overfitting and improving robustness—but also for clinical practice. Fewer required diagnostic features lower testing costs, accelerate diagnostic speed, and enhance clinical efficiency, enabling more timely therapeutic interventions. Considering the imbalance of positive and negative samples in the dataset (232:68 in the train set and 151:39 in the test set), we selected the F1 score as the evaluation metric. Metrics like AUC or ACC may not fully capture performance in such imbalanced datasets—AUC does not account for class imbalance, and accuracy can be misleading when the majority class dominates. In contrast, the F1 score balances precision and recall, providing a more reliable evaluation in this context. We also optimised hyperparameters using the average F1 score from 5‐fold cross‐validation to ensure robustness across data subsets.

In feature selection, this study employed 12 classification algorithms to address biases from using a single machine learning algorithm. Different machine learning models usually have their own feature weight analysis methods, such as the coefficients of SVM and the feature importance of Random Forest [39, 40, 41, 42]. However, these methods are mainly used for internal model analysis and are not suitable for comparing feature importance between algorithms, which may introduce bias. To overcome this, we applied two external evaluation techniques: SHAP and PI. SHAP is based on the Shapley value in game theory and quantifies the contribution of features to individual predictions and overall predictions. PI calculates feature importance by randomly shuffling the order of features and evaluating the decrease in performance scores. By using these two independent feature evaluation methods, we obtained a unified perspective to evaluate the feature importance of the twelve models and finally selected the top 10 features in each model. Then, the number of features was gradually reduced from 10 to 5, with hyperparameter optimisation and retraining after each step. Internal and external validations showed that the AdaBoost Classifier with 5 features performed best, achieving F1 scores of 0.9016 (SHAP) and 0.9063 (PI) on the test set, surpassing the 32‐feature model (F1 = 0.8652). In addition, by comparing the scatter plot between the train set and the test set, it can be seen that the 32‐feature model achieved almost perfect classification on the train set, but its performance on the test set was far from that on the train set, and overfitting occurred. In contrast, the feature‐selected model achieved more consistent predictions across the train and test sets, significantly improving generalisation.

We found that after reducing the number of features from 32 to 5, the model performance not only did not degrade but improved, and the generalisation ability was also better, suggesting that the importance of features may have long‐tailed distributions, that is, a small number of key features dominate model predictions, while most features have limited marginal contributions. The likely cause is strong correlations or redundancy between features, enabling the model to reconstruct key information from the remaining features and maintain performance despite feature reduction. At the same time, we found that in the process of feature selection using the PI method, the AdaBoost Classifier models with 5 and 6 features both obtained the highest F1 score of 0.9063 on the test set. Among them, the 5‐feature model removed the PLT feature compared to the 6‐feature model. The analysis by calculating the Pearson correlation coefficient showed that PLT was not significantly correlated with other features in the train set, but it showed a strong correlation with total bilirubin (TBIL), direct bilirubin (DBIL) and AST features in the test set. This phenomenon may stem from differences in data distribution between the test and train sets due to their origin from different centres. Our feature selection method demonstrates that in artificial intelligence model development, more features do not necessarily lead to better performance. While high‐dimensional data provide abundant information, not all features enhance model performance. Redundant features or noise may even reduce generalisability. In modelling medical data, the importance of the selected features should be fully considered to optimise model performance and improve its robustness.

We found that PCT, PLT and AST are key markers significantly linked to HIV with OIs. Whether based on SHAP or PI, these three features ranked very highly across the twelve models, frequently appearing in the top ten. PCT, a sensitive inflammatory marker, surpasses CRP in detecting bacterial infections [43]. In HIV patients, immune activation elevates IL‐6, which stimulates PCT expression [44, 45]. Phatlhane et al. observed that PCT levels typically remain unchanged in asymptomatic, untreated HIV patients, only increasing in the presence of significant bacterial infections, consistent with the findings of this study [46]. HIV infection suppresses bone marrow haematopoiesis, resulting in reductions in HB and PLT, which weaken immune function and heighten the risk of OIs [47, 48]. Moreover, OI pathogens can directly invade bone marrow and vasculature, further reducing HB and PLT levels. Notably, the AST feature ranked third overall but was not included in the optimal model's five features. This may be due to differences in the way features are fitted in different models, resulting in AST being more important in models other than the AdaBoost Classifier. Nevertheless, AST is commonly used in clinical practice as a marker for liver function and tissue damage, and it may still have potential value in the diagnosis of OIs, particularly in identifying organ dysfunction associated with infections.

One highlight of this study is that the train and test sets come from different sources, with the train set from Wenzhou Central Hospital and the test set from four nationwide centres. Except for 75 samples from Wenzhou Central Hospital, the test set data does not overlap with the train set. This setup simulates regional distribution differences in real clinical scenarios, enabling a realistic evaluation of the model's generalisability. This design strategy enhances the external validity of model performance and aligns the findings with practical clinical needs, providing a solid foundation for cross‐regional application and promotion.

Another highlight is that we have successfully constructed an efficient and concise model that delivers excellent performance with minimal feature dimensions, maximising convenience and practicality for clinical use. However, some limitations must be acknowledged. First, as a retrospective study, certain biases are unavoidable, such as selection bias due to the non‐random selection of patients and information bias arising from incomplete or inaccurate patient records. Future multicentre randomised clinical trials with larger sample sizes are needed to confirm its clinical utility. Second, vertical HIV transmission affects 2.9% of pregnant women globally, and physiological changes during pregnancy, such as dilutional anaemia and increased renal clearance, may complicate OI diagnosis and impact model performance in this population [5, 49, 50, 51, 52].

## 5 Conclusion

This study is the first to develop and validate a machine learning diagnostic model capable of rapidly identifying whether HIV‐infected individuals have any type of OIs, rather than being limited to specific types. The model, which relies on only five clinical features, has demonstrated robust performance on a multicentre test set. It effectively assesses the risk of OIs in HIV patients, offering a rapid, efficient and cost‐effective diagnostic solution for clinical practice.

## Author Contributions

**Hao Chen:** data curation (equal), investigation (equal), writing – original draft (equal). **Fanxuan Chen:** investigation (equal), methodology (equal), visualization (equal). **Yijun Wang:** data curation (equal), investigation (equal), writing – original draft (equal). **Enna Cai:** data curation (equal), investigation (equal), writing – original draft (equal). **Wangzheng Pan:** investigation (equal), methodology (equal), visualization (equal). **Yichen Li:** investigation (equal), methodology (equal), visualization (equal). **Zefei Mo:** investigation (equal), methodology (equal), visualization (equal). **Hao Lou:** investigation (equal), methodology (equal), visualization (equal). **Chufan Ren:** investigation (equal), methodology (equal), visualization (equal). **Chenyue Dai:** investigation (equal), methodology (equal), visualization (equal). **Xingbo Shan:** investigation (equal). **Hui Ye:** investigation (equal). **Zhenwei Xu:** visualization (equal). **Pu Dong:** data curation (equal). **Han Zhou:** data curation (equal). **Shuya Xu:** data curation (equal). **Tianye Zhu:** data curation (equal). **Mingzhi Su:** data curation (equal). **Xingguo Miao:** conceptualization (equal), funding acquisition (equal), project administration (equal), supervision (equal), writing – review and editing (equal). **Xiaoqu Hu:** conceptualization (equal), funding acquisition (equal), project administration (equal), supervision (equal), writing – review and editing (equal). **Liang Hong:** conceptualization (equal), funding acquisition (equal), project administration (equal), supervision (equal), writing – review and editing (equal). **Yi Wang:** project administration (equal), supervision (equal). **Feifei Su:** conceptualization (equal), funding acquisition (equal), project administration (equal), supervision (equal), writing – review and editing (equal).

## Conflicts of Interest

The authors declare no conflicts of interest.

## Supporting information


Appendix S1.

## Data Availability

The data that support the findings of this study are available from the corresponding author on reasonable request.
