# Supplementary material for: A Machine Learning Model for Diagnosing Opportunistic Infections in HIV Patients: Broad Applicability Across Infection Types
Source: J Cell Mol Med. 2025 Mar 23;29(6):e70497. doi: 10.1111/jcmm.70497 (PMC11930644; doi:10.1111/jcmm.70497)
Supplement: Supplementary file 1 — Appendix S1. [file JCMM-29-e70497-s001.docx]

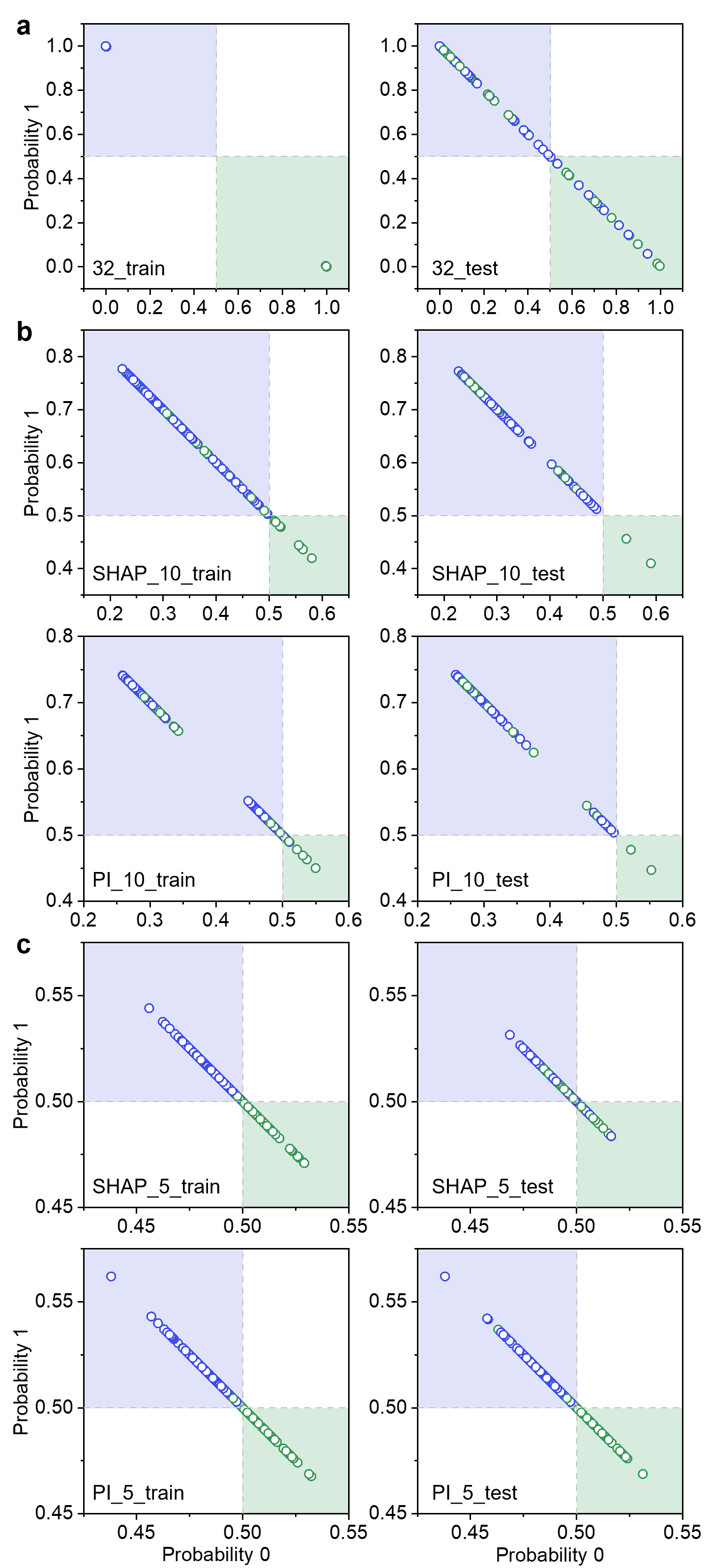


**Figure S1.** Scatter plots of the predicted probability of the best model for different number of features in the train set and test set, respectively. The vertical axis represents the probability of the model predicting a sample with Group=1, while the horizontal axis depicts the probability of the model predicting a sample with Group=0. The blue dots indicate Group 1, while the green dots indicate Group 0.


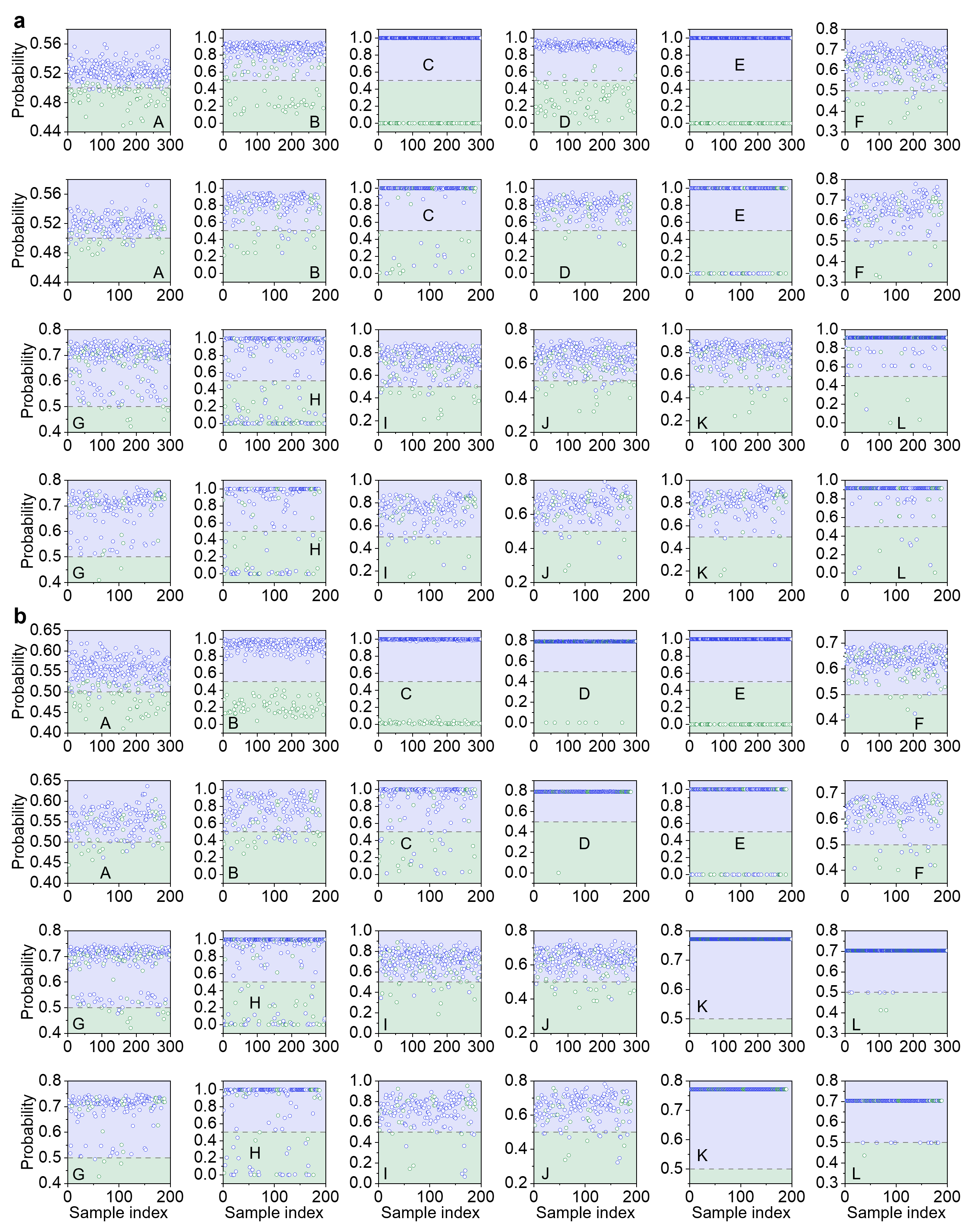


**Figure S2.** Scatterplot of the prediction probabilities of the twelve best models for the training and test set samples at 10 features. a) SHAP; b) PI.

**Table S1. Baseline Characteristics of the cohorts**

IQR, interquartile range; Percentage, the percentage shown in the figure is the proportion of each positive

*Cohort1+ Cohort2 (n=375)*

|  | **0** | **1** | **p value** |
| --- | --- | --- | --- |
|  | ***n=85*** | ***n=290*** |  |
| **Demographic Characteristics** | | | |
| Age(IQR, years) | 40.0 [31.0;54.0] | 45.0 [32.0;57.0] | 0.252 |
| Gender(Percentage) |  |  | 0.116 |
| 0 | 18 (21.2%) | 39 (13.4%) |  |
| 1 | 67 (78.8%) | 251 (86.6%) |  |
| **Clinical Characteristics**  **(Percentage)** | | | |
| Fever | 49 (57.6%) | 188 (64.8%) | 0.280 |
| Cough | 36 (42.4%) | 175 (60.3%) | 0.005 |
| Rash | 23 (27.1%) | 43 (14.8%) | 0.015 |
| **Laboratory Examinations**  **(IQR)** | | | |
| WBC(10^9^/L) | 3.90 [2.50;5.50] | 4.70 [3.20;6.60] | 0.011 |
| NEU(10^9^/L) | 2.80 [1.90;4.10] | 3.20 [2.10;5.18] | 0.059 |
| LYM(10^9^/L) | 0.60 [0.30;1.10] | 0.70 [0.40;1.08] | 0.293 |
| MONO(10^9^/L) | 0.25 [0.13;0.35] | 0.31 [0.17;0.48] | 0.010 |
| EOS(10^9^/L) | 0.05 [0.02;0.11] | 0.06 [0.02;0.18] | 0.296 |
| BASO(10^9^/L) | 0.01 [0.00;0.02] | 0.01 [0.00;0.02] | 0.156 |
| HB(g/L) | 100 [88.0;120] | 111 [94.0;126] | 0.008 |
| RBC(10^12^/L) | 3.51 [3.05;3.97] | 3.80 [3.21;4.30] | 0.005 |
| PLT(10^9^/L) | 124 [62.0;210] | 186 [121;261] | <0.001 |
| ALT(U/L) | 36.0 [21.0;68.0] | 25.0 [15.0;49.8] | 0.002 |
| AST(U/L) | 61.0 [32.0;108] | 35.0 [23.0;65.8] | <0.001 |
| CD4(10^6^/L) | 26.0 [9.00;126] | 46.5 [12.0;128] | 0.296 |
| LDH(U/L) | 294 [205;477] | 272 [194;448] | 0.320 |
| D-dimer(mg/L) | 1.23 [0.39;3.53] | 0.71 [0.30;2.60] | 0.097 |
| CRP(mg/L) | 40.8 [6.60;80.8] | 38.8 [13.3;74.6] | 0.911 |
| TBIL(µmol/L) | 11.2 [7.40;17.6] | 9.15 [6.80;13.2] | 0.020 |
| DBIL(µmol/L) | 4.10 [2.50;7.90] | 3.60 [2.42;5.68] | 0.149 |
| IBIL(µmol/L) | 5.90 [4.60;9.00] | 5.15 [3.73;7.88] | 0.036 |
| ALB(mg/L) | 31.5 [25.1;36.3] | 32.1 [27.5;36.5] | 0.361 |
| Urea(mmol/L) | 4.10 [3.00;5.50] | 4.30 [3.10;5.60] | 0.814 |
| CRE(µmol/L) | 66.0 [57.0;80.0] | 68.0 [55.0;82.0] | 0.622 |
| K+(mmol/L) | 3.81 [3.54;4.01] | 3.91 [3.59;4.18] | 0.114 |
| PT(s) | 12.5 [11.4;13.2] | 12.2 [11.5;13.2] | 0.688 |
| CD4/CD8 | 0.08 [0.04;0.21] | 0.10 [0.04;0.27] | 0.417 |
| ALP(U/L) | 92.0 [69.0;126] | 85.0 [63.0;129] | 0.359 |
| γ-GT(U/L) | 59.0 [37.0;111] | 52.0 [31.0;126] | 0.303 |
| PCT(ng/mL) | 1.04 [0.19;2.63] | 0.38 [0.11;1.28] | <0.001 |

*Cohort3(n=115)*

|  | **0** | **1** | **p value** |
| --- | --- | --- | --- |
|  | ***n=22*** | ***n=93*** |  |
| **Demographic Characteristics** | | | |
| Age(IQR, years) | 33.5 [29.2;45.2] | 33.0 [29.0;45.0] | 0.890 |
| Gender(Percentage) |  |  | 0.401 |
| 0 | 3 (13.6%) | 7 (7.53%) |  |
| 1 | 19 (86.4%) | 86 (92.5%) |  |
| **Clinical Characteristics**  **(Percentage)** | | | |
| Fever | 14 (63.6%) | 49 (52.7%) | 0.490 |
| Cough | 19 (86.4%) | 38 (40.9%) | <0.001 |
| Rash | 6 (27.3%) | 34 (36.6%) | 0.566 |
| **Laboratory Examinations**  **(IQR)** | | | |
| WBC(10^9^/L) | 4.58 [3.40;7.27] | 3.90 [2.72;5.07] | 0.082 |
| NEU(10^9^/L) | 3.36 [2.40;5.95] | 2.65 [1.88;3.90] | 0.036 |
| LYM(10^9^/L) | 0.42 [0.25;0.74] | 0.57 [0.27;0.89] | 0.337 |
| MONO(10^9^/L) | 0.30 [0.12;0.47] | 0.27 [0.14;0.41] | 0.859 |
| EOS(10^9^/L) | 0.04 [0.01;0.16] | 0.04 [0.01;0.14] | 0.816 |
| BASO(10^9^/L) | 0.01 [0.00;0.02] | 0.01 [0.01;0.02] | 0.179 |
| HB(g/L) | 93.0 [81.0;113] | 109 [86.0;123] | 0.167 |
| RBC(10^12^/L) | 3.44 [2.97;3.91] | 3.75 [2.86;4.22] | 0.447 |
| PLT(10^9^/L) | 174 [130;243] | 176 [109;273] | 0.986 |
| ALT(U/L) | 32.0 [16.2;65.2] | 22.0 [15.0;49.0] | 0.332 |
| AST(U/L) | 51.5 [34.2;73.5] | 29.0 [21.0;51.0] | 0.012 |
| CD4(10^6^/L) | 16.5 [7.25;87.2] | 13.0 [6.00;27.0] | 0.183 |
| LDH(U/L) | 316 [286;419] | 278 [200;517] | 0.138 |
| D-dimer(mg/L) | 5.83 [2.50;5.83] | 2.35 [1.24;7.93] | 0.088 |
| CRP(mg/L) | 50.7 [16.4;71.0] | 27.9 [6.18;64.3] | 0.149 |
| TBIL(µmol/L) | 9.45 [5.97;11.6] | 8.30 [5.74;13.5] | 0.393 |
| DBIL(µmol/L) | 4.73 [2.85;6.46] | 4.10 [2.90;6.30] | 0.518 |
| IBIL(µmol/L) | 4.15 [3.10;5.25] | 3.70 [2.40;4.80] | 0.163 |
| ALB(mg/L) | 29.5 [24.6;36.3] | 32.9 [28.3;39.3] | 0.128 |
| Urea(mmol/L) | 4.90 [3.10;6.48] | 4.16 [3.38;5.41] | 0.296 |
| CRE(µmol/L) | 72.5 [57.2;84.8] | 72.0 [58.0;83.0] | 0.946 |
| K+(mmol/L) | 3.84 [3.70;3.97] | 3.91 [3.64;4.30] | 0.565 |
| PT(s) | 13.6 [12.3;13.7] | 12.8 [11.9;14.0] | 0.384 |
| CD4/CD8 | 0.10 [0.03;0.29] | 0.04 [0.02;0.09] | 0.017 |
| ALP(U/L) | 110 [81.0;167] | 95.0 [69.0;136] | 0.166 |
| γ-GT(U/L) | 55.0 [33.0;109] | 68.0 [37.0;111] | 0.460 |
| PCT(ng/mL) | 0.32 [0.14;1.57] | 0.21 [0.07;1.20] | 0.258 |

feature in each group. Categorical variables are shown as frequencies (%), and continuous variables are shown as medians (interquartile ranges).

WBC, white blood cell; NEU, neutrophil; LYM, lymphocyte; MONO, monocyte; EOS, eosinophil; BASO, basophil; HB, haemoglobin; RBC, red blood cell; PLT, blood platelet; ALT, alanine aminotransferase; AST, aspartate aminotransferase; CD4, CD4+ T cells; LDH, lactic dehydrogenase; CRP, C-reactive protein; TBIL, total bilirubin; DBIL, direct bilirubin; IBIL, indirect bilirubin; ALB, albumin; Urea, urea nitrogen; CRE, creatinine; K+, serum potassium; PT ,prothrombin Time; ALP, alkaline phosphatase; γ-GT,γ-glutamyl transpeptidase; PCT, procalcitonin.

**Table S2.32 Features**

| Incorporating indicators | | Related Literature |
| --- | --- | --- |
| Total | Part |  |
| Age  Gender  Fever  Cough  Rash  WBC (10^9^/L)  NEU (10^9^/L)  LYM (10^9^/L)  MONO (10^9^/L)  EOS (10^9^/L)  BASO (10^9^/L)  HB (g/L)  RBC (10^12^/L)  PLT (10^9^/L)  ALT (U/L)  AST (U/L)  CD4 (10^6^/L)  LDH (U/L)  D-dimer (mg/L)  CRP (mg/L)  TBIL (µmol/L)  DBIL (µmol/L)  IBIL (µmol/L)  ALB (g/L)  Urea (mmol/L)  CRE (µmol/L)  K+ (mmol/L)  PT (s）  CD4/CD8  ALP (U/L)  γ-GT (U/L)  PCT (ng/mL) | Age, Gender | HIV and tuberculosis co-infection in a highly HIV-infected population of rivers state, Nigeria^1^ |
|  | Cough, Fever, CRP, ALB, HB | What do the clinical features of positive nontuberculous mycobacteria isolates from patients with HIV/AIDS in China reveal? A systematic review and meta-analysis^2^ |
|  | Rash | Skin and Mucocutaneous Manifestations: Useful Clinical Predictors of HIV/AIDS^3^ |
|  | NEU | Following successful anti-leishmanial treatment, neutrophil counts, CD10 expression and phagocytic capacity remain reduced in visceral leishmaniasis patients co-infected with HIV^4^ |
|  | NEU, CD4 | Differences in the clinical characteristics of Pneumocystis jirovecii pneumonia in immunocompromized patients with and without HIV infection^5^ |
|  | LYM | The use of total lymphocyte count as a surrogate for low CD4+ T lymphocyte cell counts among HIV-1-infected women in Tanzania^6^ |
|  | MONO | Monocyte activation in persons living with HIV and tuberculosis coinfection^7^ |
|  | EOS | Eosinophilia: clinical significance in HIV-infected individuals^8^ |
|  | BASO, EOS | Leucocytes and Th-associated Cytokine Profile of HIV-Leishmaniasis Co-Infected Persons Attending Abuja Teaching Hospital, Nigeria^9^ |
|  | HB, CD4, PLT, LDH, Urea | Multi-Clinical Factors Combined with an Artificial Intelligence Algorithm Diagnosis Model for HIV-Infected People with Bloodstream Infection^10^ |
|  | HB, K+ | Comparison of clinical features in patients with persistent and nonpersistent cryptococcal meningitis: twelve years of clinical experience in four centers in China^11^ |
|  | RBC | Malaria parasite prevalence and Haematological parameters in HIV seropositive patients attending the regional hospital Limbe, Cameroon: a hospital-based cross-sectional study^12^ |
|  | PLT | HCV coinfection aggravated the decrease of platelet counts, but not mean platelet volume in chronic HIV-infected patients^13^ |
|  | ALT, AST | Clinical and virological characteristics of hepatitis B or C virus co-infection with HIV in Indonesian patients^14^ |
|  | AST, ALT | Characterization of HIV–HBV coinfection in a multinational HIV-infected cohort^15^ |
|  | CD4 | Cytomegalovirus (CMV) infection in HIV/AIDS patients and diagnostic values of CMV-DNA detection across different sample types^16^ |
|  | CD4, Fever, Rash, HB, PLT, WBC, ALT, AST, ALP, TBIL | Clinical features of HIV positive talaromycosis marneffei patients and development of a risk prediction model^17^ |
|  | LDH | (1–3)-Beta-D-glucan in association with lactate dehydrogenase as biomarkers of Pneumocystis pneumonia (PcP) in HIV-infected patients^18^ |
|  | D-dimer | Factors associated with D-dimer levels in HIV-infected individuals^19^ |
|  | CRP,PCT | C-reactive protein and procalcitonin to discriminate between tuberculosis, Pneumocystis jirovecii pneumonia, and bacterial pneumonia in HIV-infected inpatients meeting WHO criteria for seriously ill: a prospective cohort study^20^ |
|  | TBIL | HCV Genotype Distribution and Clinical Characteristics of HCV Mono-Infected and HCV/HIV Co-Infected Patients in Liangshan Prefecture, Sichuan Province, China^21^ |
|  | DBIL | LIVER FUNCTION MARKERS AND ASSOCIATED SERUM ELECTROLYTES CHANGES IN HIV PATIENTS ATTENDING PATIENT SUPPORT CENTRE OF JARAMOGI OGINGA ODINGA TEACHING AND REFERRAL HOSPITAL, KISUMU COUNTY, KENYA^22^ |
|  | DBIL | [The blood biochemical indicators in drug addicted patients with HIV-infection and viral hepatitis] ^23^ |
|  | IBIL, DBIL, CD4, ALB | Epidemiology of Hepatitis C Virus in HIV Patients from West Mexico: Implications for Controlling and Preventing Viral Hepatitis^24^ |
|  | ALB | Association of serum albumin and aspartate transaminase with 5-year all-cause mortality in HIV/hepatitis C virus coinfection and HIV monoinfection^25^ |
|  | CRE, AST, ALT, γ-GT | Inflammatory status hepatic enzymes and serum creatinine in HIV-, HIV+ and HIV-TB co-infected adult Central Africans^26^ |
|  | PT | Gastrointestinal disseminated histoplasmosis in HIV-infected patients: A descriptive and comparative study^27^ |
|  | CD4/CD8 | Clinical symptoms and immune injury reflected by low CD4/CD8 ratio should increase the suspicion of HIV coinfection with tuberculosis^28^ |

**Table S3. Feature Distribution Analysis**

*Training vs. Test Set*

| **Feature** | **Test** | **p-value** | **Significant** |
| --- | --- | --- | --- |
| Gender | Chi-Square | 0.080 | No |
| Fever | Chi-Square | 0.974 | No |
| Cough | Chi-Square | 0.393 | No |
| Rash | Chi-Square | 0.001 | Yes |
| Age | Mann-Whitney U | <0.001 | Yes |
| WBC | Mann-Whitney U | 0.030 | Yes |
| NEU | Mann-Whitney U | 0.090 | No |
| LYM | Mann-Whitney U | 0.003 | Yes |
| MONO | Mann-Whitney U | 0.043 | Yes |
| EOS | Mann-Whitney U | 0.059 | No |
| BASO | Mann-Whitney U | 0.806 | No |
| HB | Mann-Whitney U | 0.074 | No |
| RBC | Mann-Whitney U | 0.171 | No |
| PLT | Mann-Whitney U | 0.828 | No |
| ALT | Mann-Whitney U | 0.688 | No |
| AST | Mann-Whitney U | 0.598 | No |
| CD4 | Mann-Whitney U | <0.001 | Yes |
| LDH | Mann-Whitney U | 0.052 | No |
| D-dimer | Mann-Whitney U | <0.001 | Yes |
| CRP | Mann-Whitney U | 0.830 | No |
| TBIL | Mann-Whitney U | 0.130 | No |
| DBIL | Mann-Whitney U | 0.006 | Yes |
| IBIL | Mann-Whitney U | <0.001 | Yes |
| ALB | Mann-Whitney U | 0.791 | No |
| Urea | Mann-Whitney U | 0.438 | No |
| CRE | Mann-Whitney U | 0.560 | No |
| K+ | Mann-Whitney U | 0.754 | No |
| PT | Mann-Whitney U | 0.001 | Yes |
| CD4/CD8 | Mann-Whitney U | <0.001 | Yes |
| ALP | Mann-Whitney U | 0.059 | No |
| γ-GT | Mann-Whitney U | 0.049 | Yes |
| PCT | Mann-Whitney U | 0.119 | No |

*Intra-Training Set*

| **Feature** | **Test** | **p-value** | **Significant** |
| --- | --- | --- | --- |
| Gender | Chi-Square | 0.082 | No |
| Fever | Chi-Square | 0.576 | No |
| Cough | Chi-Square | 0.021 | Yes |
| Rash | Chi-Square | 0.044 | Yes |
| Age | Mann-Whitney U | 0.686 | No |
| WBC | Mann-Whitney U | 0.034 | Yes |
| NEU | Mann-Whitney U | 0.085 | No |
| LYM | Mann-Whitney U | 0.668 | No |
| MONO | Mann-Whitney U | 0.035 | Yes |
| EOS | Mann-Whitney U | 0.467 | No |
| BASO | Mann-Whitney U | 0.318 | No |
| HB | Mann-Whitney U | 0.019 | Yes |
| RBC | Mann-Whitney U | 0.015 | Yes |
| PLT | Mann-Whitney U | <0.001 | Yes |
| ALT | Mann-Whitney U | 0.021 | Yes |
| AST | Mann-Whitney U | <0.001 | Yes |
| CD4 | Mann-Whitney U | 0.513 | No |
| LDH | Mann-Whitney U | 0.473 | No |
| D-dimer | Mann-Whitney U | 0.225 | No |
| CRP | Mann-Whitney U | 0.705 | No |
| TBIL | Mann-Whitney U | 0.038 | Yes |
| DBIL | Mann-Whitney U | 0.456 | No |
| IBIL | Mann-Whitney U | 0.022 | Yes |
| ALB | Mann-Whitney U | 0.134 | No |
| Urea | Mann-Whitney U | 0.685 | No |
| CRE | Mann-Whitney U | 0.695 | No |
| K+ | Mann-Whitney U | 0.063 | No |
| PT | Mann-Whitney U | 0.784 | No |
| CD4/CD8 | Mann-Whitney U | 0.758 | No |
| ALP | Mann-Whitney U | 0.614 | No |
| γ-GT | Mann-Whitney U | 0.664 | No |
| PCT | Mann-Whitney U | <0.001 | Yes |

*Intra-Training Set*

| **Feature** | **Test** | **p-value** | **Significant** |
| --- | --- | --- | --- |
| Gender | Chi-Square | 0.719 | No |
| Fever | Chi-Square | 1.000 | No |
| Cough | Chi-Square | 0.012 | Yes |
| Rash | Chi-Square | 1.000 | No |
| Age | Mann-Whitney U | 0.324 | No |
| WBC | Mann-Whitney U | 0.740 | No |
| NEU | Mann-Whitney U | 0.270 | No |
| LYM | Mann-Whitney U | 0.084 | No |
| MONO | Mann-Whitney U | 0.341 | No |
| EOS | Mann-Whitney U | 0.794 | No |
| BASO | Mann-Whitney U | 0.099 | No |
| HB | Mann-Whitney U | 0.048 | Yes |
| RBC | Mann-Whitney U | 0.146 | No |
| PLT | Mann-Whitney U | 0.671 | No |
| ALT | Mann-Whitney U | 0.017 | Yes |
| AST | Mann-Whitney U | 0.002 | Yes |
| CD4 | Mann-Whitney U | 0.626 | No |
| LDH | Mann-Whitney U | 0.143 | No |
| D-dimer | Mann-Whitney U | 0.039 | Yes |
| CRP | Mann-Whitney U | 0.085 | No |
| TBIL | Mann-Whitney U | 0.176 | No |
| DBIL | Mann-Whitney U | 0.116 | No |
| IBIL | Mann-Whitney U | 0.189 | No |
| ALB | Mann-Whitney U | 0.248 | No |
| Urea | Mann-Whitney U | 0.901 | No |
| CRE | Mann-Whitney U | 0.831 | No |
| K+ | Mann-Whitney U | 0.672 | No |
| PT | Mann-Whitney U | 0.252 | No |
| CD4/CD8 | Mann-Whitney U | 0.180 | No |
| ALP | Mann-Whitney U | 0.079 | No |
| γ-GT | Mann-Whitney U | 0.693 | No |
| PCT | Mann-Whitney U | 0.073 | No |

References

1. Okonko IO, Anyanwu A, U Osadebe A, N Odu N. HIV and tuberculosis co-infection in a highly HIV-infected population of rivers state, Nigeria. *J Immunoassay Immunochem*. 2018;39(6):636-646. doi: 10.1080/15321819.2018.1529681
2. Yuan J, Wang Y, Wang L, Wang H, Ren Y, Yang W. What do the clinical features of positive nontuberculous mycobacteria isolates from patients with HIV/AIDS in China reveal? A systematic review and meta-analysis. *J Glob Health*. 2023;13:04093. doi: 10.7189/jogh.13.04093
3. Chopra S, Arora U. Skin and Mucocutaneous Manifestations: Useful Clinical Predictors of HIV/AIDS. *J Clin Diagn Res JCDR*. 2012;6(10):1695-1698. doi: 10.7860/JCDR/2012/4615.2633
4. Takele Y, Adem E, Mulaw T, Müller I, Cotton JA, Kropf P. Following successful anti-leishmanial treatment, neutrophil counts, CD10 expression and phagocytic capacity remain reduced in visceral leishmaniasis patients co-infected with HIV. *PLoS Negl Trop Dis*. 2022;16(8):e0010681. doi: 10.1371/journal.pntd.0010681
5. Enomoto T, Azuma A, Kohno A, et al. Differences in the clinical characteristics of Pneumocystis jirovecii pneumonia in immunocompromized patients with and without HIV infection. *Respirol Carlton Vic*. 2010;15(1):126-131. doi: 10.1111/j.1440-1843.2009.01660.x
6. Mgomella GS, Venkatesh PA, Bosch RJ, et al. The use of total lymphocyte count as a surrogate for low CD4+ T lymphocyte cell counts among HIV-1-infected women in Tanzania. *East Afr J Public Health*. 2010;7(2):160-164.
7. Huaman MA, Juchnowski SM, Zidar DA, et al. Monocyte activation in persons living with HIV and tuberculosis coinfection. *AIDS Lond Engl*. 2021;35(3):447-452. doi: 10.1097/QAD.0000000000002766
8. Sivaram M, White A, Radcliffe KW. Eosinophilia: clinical significance in HIV-infected individuals. *Int J STD AIDS*. 2012;23(9):635-638. doi: 10.1258/ijsa.2012.011409
9. Abdullahi IN, Emeribe AU, Adekola HA, et al. Leucocytes and Th-associated Cytokine Profile of HIV-Leishmaniasis Co-Infected Persons Attending Abuja Teaching Hospital, Nigeria. *Eurasian J Med*. 2020;52(3):271-276. doi: 10.5152/eurasianjmed.2020.20008
10. Wu L, Xia D, Xu K. Multi-Clinical Factors Combined with an Artificial Intelligence Algorithm Diagnosis Model for HIV-Infected People with Bloodstream Infection. *Infect Drug Resist*. 2023;16:6085-6097. doi: 10.2147/IDR.S423709
11. Xu XG, Pan WH, Bi XL, et al. Comparison of clinical features in patients with persistent and nonpersistent cryptococcal meningitis: twelve years of clinical experience in four centers in China. *CNS Neurosci Ther*. 2013;19(8):625-631. doi: 10.1111/cns.12135
12. Sandie SM, Sumbele IUN, Tasah MM, Kimbi HK. Malaria parasite prevalence and Haematological parameters in HIV seropositive patients attending the regional hospital Limbe, Cameroon: a hospital-based cross-sectional study. *BMC Infect Dis*. 2019;19(1):988. doi: 10.1186/s12879-019-4629-4
13. Lv L, Li Y, Fan X, Xie Z, Liang H, Shen T. HCV coinfection aggravated the decrease of platelet counts, but not mean platelet volume in chronic HIV-infected patients. *Sci Rep*. 2018;8(1):17497. doi: 10.1038/s41598-018-35705-9
14. Anggorowati N, Yano Y, Heriyanto DS, et al. Clinical and virological characteristics of hepatitis B or C virus co-infection with HIV in Indonesian patients. *J Med Virol*. 2012;84(6):857-865. doi: 10.1002/jmv.23293
15. Thio CL, Smeaton L, Saulynas M, et al. Characterization of HIV-HBV coinfection in a multinational HIV-infected cohort. *AIDS Lond Engl*. 2013;27(2):191-201. doi: 10.1097/QAD.0b013e32835a9984
16. Zhao M, Zhuo C, Li Q, Liu L. Cytomegalovirus (CMV) infection in HIV/AIDS patients and diagnostic values of CMV-DNA detection across different sample types. *Ann Palliat Med*. 2020;9(5):2710-2715. doi: 10.21037/apm-20-1352
17. Cen J, Huang J, Zeng W, Pan M, Qiu Y, Zhang J. Clinical features of HIV positive talaromycosis marneffei patients and development of a risk prediction model. *Heliyon*. 2023;9(9):e20068. doi: 10.1016/j.heliyon.2023.e20068
18. Esteves F, Lee CH, de Sousa B, et al. (1-3)-beta-D-glucan in association with lactate dehydrogenase as biomarkers of Pneumocystis pneumonia (PcP) in HIV-infected patients. *Eur J Clin Microbiol Infect Dis Off Publ Eur Soc Clin Microbiol*. 2014;33(7):1173-1180. doi: 10.1007/s10096-014-2054-6
19. Borges AH, O’Connor JL, Phillips AN, et al. Factors associated with D-dimer levels in HIV-infected individuals. *PloS One*. 2014;9(3):e90978. doi: 10.1371/journal.pone.0090978
20. Mendelson F, Griesel R, Tiffin N, et al. C-reactive protein and procalcitonin to discriminate between tuberculosis, Pneumocystis jirovecii pneumonia, and bacterial pneumonia in HIV-infected inpatients meeting WHO criteria for seriously ill: a prospective cohort study. *BMC Infect Dis*. 2018;18(1):399. doi: 10.1186/s12879-018-3303-6
21. Cao B, Liu M, Jiang T, et al. HCV Genotype Distribution and Clinical Characteristics of HCV Mono-Infected and HCV/HIV Co-Infected Patients in Liangshan Prefecture, Sichuan Province, China. *J Int Assoc Provid AIDS Care*. 2023;22:23259582231217810. doi: 10.1177/23259582231217810
22. Opiyo WO, Ng’Wena AGM, Ofulla AVO. LIVER FUNCTION MARKERS AND ASSOCIATED SERUM ELECTROLYTES CHANGES IN HIV PATIENTS ATTENDING PATIENT SUPPORT CENTRE OF JARAMOGI OGINGA ODINGA TEACHING AND REFERRAL HOSPITAL, KISUMU COUNTY, KENYA. *East Afr Med J*. 2013;90(9):276-287.
23. Korshunov GV, Bychkov EN, Arsent’eva LA, Serkova SA, Borodulin VB. [The blood biochemical indicators in drug addicted patients with HIV-infection and viral hepatitis]. *Klin Lab Diagn*. 2012;(7):17-18.
24. Jose-Abrego A, Trujillo-Trujillo ME, Laguna-Meraz S, Roman S, Panduro A. Epidemiology of Hepatitis C Virus in HIV Patients from West Mexico: Implications for Controlling and Preventing Viral Hepatitis. *Pathog Basel Switz*. 2024;13(5). doi: 10.3390/pathogens13050360
25. Scherzer R, Heymsfield SB, Rimland D, et al. Association of serum albumin and aspartate transaminase with 5-year all-cause mortality in HIV/hepatitis C virus coinfection and HIV monoinfection. *AIDS Lond Engl*. 2017;31(1):71-79. doi: 10.1097/QAD.0000000000001278
26. Mokondjimobe E, Longo-Mbenza B, Mampouya-Arrouse P, Parra HJ, Diatewa M. Inflammatory status hepatic enzymes and serum creatinine in HIV-, HIV+ and HIV-TB co-infected adult Central Africans. *Int J Gen Med*. 2012;5:961-965. doi: 10.2147/IJGM.S31199
27. Nacher M, Valdes A, Adenis A, et al. Gastrointestinal disseminated histoplasmosis in HIV-infected patients: A descriptive and comparative study. *PLoS Negl Trop Dis*. 2021;15(1):e0009050. doi: 10.1371/journal.pntd.0009050
28. Li L, Abudureheman Z, Zhong X, et al. Clinical symptoms and immune injury reflected by low CD4/CD8 ratio should increase the suspicion of HIV coinfection with tuberculosis. *Heliyon*. 2023;9(3):e14219. doi: 10.1016/j.heliyon.2023.e14219
